# Viral Diseases in Dermatology

**DOI:** 10.3390/v15020513

**Published:** 2023-02-13

**Authors:** Mohamad Goldust

**Affiliations:** Department of Dermatology, Yale University School of Medicine, New Haven, CT 06510, USA; mohamad.goldust@yale.edu

Viral skin infections are some of the most common skin diseases in medical dermatology. Despite all the advances in the understanding of these disorders, the viruses that lead to common skin infections continue to evade complete destruction. In this context, the editors of the journal *Viruses* decided to create a Special Issue, entitled “Viral Diseases in Dermatology”. It encompasses six articles that cover various aspects of viral skin diseases [1,2,3,4,5,6].

This Special Issue sheds light on various aspects of viral diseases in dermatology, including, but not limited to, pathophysiology and treatment. To be brief, the key published articles in this Special Issue addressed the following topics: skin herpes viruses and their manifestations as well as management [1,2]; viral triggers for endemic pemphigus foliaceus [3]; and the etiological characterization of skin warts [4]. Additionally, the mechanisms and management of skin lesions in COVID-19 were highlighted in two articles [5,6]. 

Special thanks go to esteemed scientists from across the globe who contributed to this Special Issue for their immeasurable dedication and support.

Let us hope that, with new discoveries in the pathophysiology and management of viral skin diseases, we can have better treatment options with which to combat these infectious disorders.
